# Comparing performance measures and clinical outcomes between mobile stroke units and usual care in underserved areas

**DOI:** 10.1007/s10072-022-06550-6

**Published:** 2022-12-14

**Authors:** Yongchai Nilanont, Pornchai Chanyagorn, Karuna Shukij, Waitayaporn Pengtong, Mananchaya Kongmuangpuk, Kanokkarn Wongmayurachat, Kittiya Nittayaboon, Yodchanan Wongsawat, Ronnachai Sirovetnukul, Tipa Chakorn, Sattha Riyapan, Chitapa Kaveeta, Songkram Chotik-anuchit, Trongtum Tongdee, Ploypailin Thabmontian, Porntep Saeheng, Cherdchai Nopmaneejumruslers, Visit Vamvanij

**Affiliations:** 1 Division of Neurology, Department of Medicine, Faculty of Medicine Siriraj Hospital, Mahidol University, Bangkok, Thailand; 2 Siriraj Stroke Center, Faculty of Medicine Siriraj Hospital, Mahidol University, Bangkok, Thailand; 3 Department of Electrical Engineering, Faculty of Engineering, Mahidol University, Nakhon Pathom, Thailand; 4 Department of Biomedical Engineering, Faculty of Engineering, Mahidol University, Nakhon Pathom, Thailand; 5 Department of Industrial Engineering, Faculty of Engineering, Mahidol University, Nakhon Pathom, Thailand; 6 Department of Emergency Medicine, Faculty of Medicine Siriraj Hospital, Mahidol University, Bangkok, Thailand; 7 Department of Radiology, Faculty of Medicine Siriraj Hospital, Mahidol University, Bangkok, Thailand; 8 Bangkok Emergency Medical Service, Medical Service Department, Bangkok, Thailand; 9 Office of the Director of Siriraj Hospital, Faculty of Medicine Siriraj Hospital, Mahidol University, Bangkok, Thailand

**Keywords:** Performance measures, Stroke care, Underserved areas, Mobile stroke units, Reperfusion therapies

## Abstract

**Background:**

The efficacy of mobile stroke units (MSUs) in improving acute ischemic stroke (AIS) care in developing countries is unknown. We compared performance measures and stroke outcomes in AIS patients between MSU and usual care: emergency medical services (EMS) and walk-in.

**Methods:**

We enrolled patients > 18 years of age with an AIS within 4.5 h after onset. Demographic data, types, and time of reperfusion therapies and clinical outcomes were recorded. A favorable outcome was defined as a modified Rankin Scale (mRS) 0–2 at 3 months.

**Results:**

A total of 978 AIS patients (MSU = 243, EMS = 214, walk-in = 521) were enrolled between June 1, 2018, and April 30, 2021. The mean age (± SD) was 66 (± 14) years, and 510 (52.1%) were male. AIS time metrics were the shortest in the MSU with a mean (± SD) door to needle (DN) time of 20 (± 7), 29 (± 13), and 35 (± 16) min (*p* < 0.001) and door to puncture (DP) time of 73 ± 19, 86 ± 33, and 101 ± 42 min (*p* < 0.001) in MSU, EMS, and walk-in, respectively. Participants in the MSU (56.8%) received higher rate of reperfusion therapie(s) when compared to the EMS (51.4%) and walk-in (31.5%) (*p* < 0.001). After adjustment for any potential confounders and using the EMS as a reference, the MSU has the highest likelihood of achieving a favorable outcome (adjusted OR 2.15; 95% CI 1.39–3.32).

**Conclusions:**

In underserved populations, MSUs significantly reduced DN time, increased the likelihood of receiving reperfusion treatment, and achieved independency at 3 months when compared to usual care.

## Background

Developing countries represent the major burden of stroke worldwide, accounting for 75.2% of all stroke-related deaths and 81.0% of the associated disability-adjusted life years (DALYs) lost [1]. In addition, given that 60% of the world’s population reside in Asia (mostly living in developing countries), accessibility to acute stroke therapies is a growing concern among this region [2, 3]. The complexities associated with effective solutions to facilitate reperfusion therapies include the understanding of conflicting scenarios: small distant communities (e.g., remote or rural areas) and big cities (those who are close in distance but affected by delays caused by heavy traffic) [4]. There are different practices across countries regarding reperfusion therapies. International studies illustrated the most common factors associated with therapeutic decisions even when there are no specific guidelines. Overall, most investigators are applying current guidelines to optimize delivery of reperfusion therapies [5–7]. However, the existing main solutions (e.g., telestroke and regionalization of stroke care) have practical limitations to effectively delivering reperfusion therapies [3]. A paradigm change is required by targeting solutions according to the location of the stroke case index (e.g., effective access to acute stroke therapies in populated and remote areas in developing countries). Recent studies in developed countries from Germany and the USA showed that mobile stroke units (MSUs) shorten the time to reperfusion treatment and reduce post-stroke disabilities at 3 months [8–10]. However, the evidence of MSUs operation in underserved areas from developing countries is lacking.

In the present study, we evaluated and compared key time process measures, accessibility to acute reperfusion therapies, and clinical outcomes of MSUs with usual care (arrival to the emergency department by emergency medical services (EMS) and walk-in) in a large referral stroke center in Bangkok, Thailand (Siriraj Hospital, Mahidol University).

## Methods

### Study design and participants

This was a prospective proof-of-concept study conducted at the Siriraj Stroke Center, Siriraj Hospital, Mahidol University, Bangkok, Thailand. Patients with a diagnosis of an acute ischemic stroke (AIS) aged 18 years and older were consecutively enrolled in the study at the time of the activation of the code stroke protocol either in the emergency department (ED) or at the MSU within 4.5 h from symptom onset between June 1, 2018, and April 30, 2021. All services operate 24 h a day, 7 days a week (24/7). Participants were excluded if they presented beyond the time period, patients declared palliative, arrivals from long-term care facilities, and inter-hospital transfers.

The rationale behind the study design was that MSUs were not standard of care by 2018 and the impracticalities of conducting an RCT in a large population from an underserved area concerning delays of receiving stroke care for some patients depending on the accessibility to a tertiary stroke center.

### Healthcare system in Bangkok

**Fig. 1:**
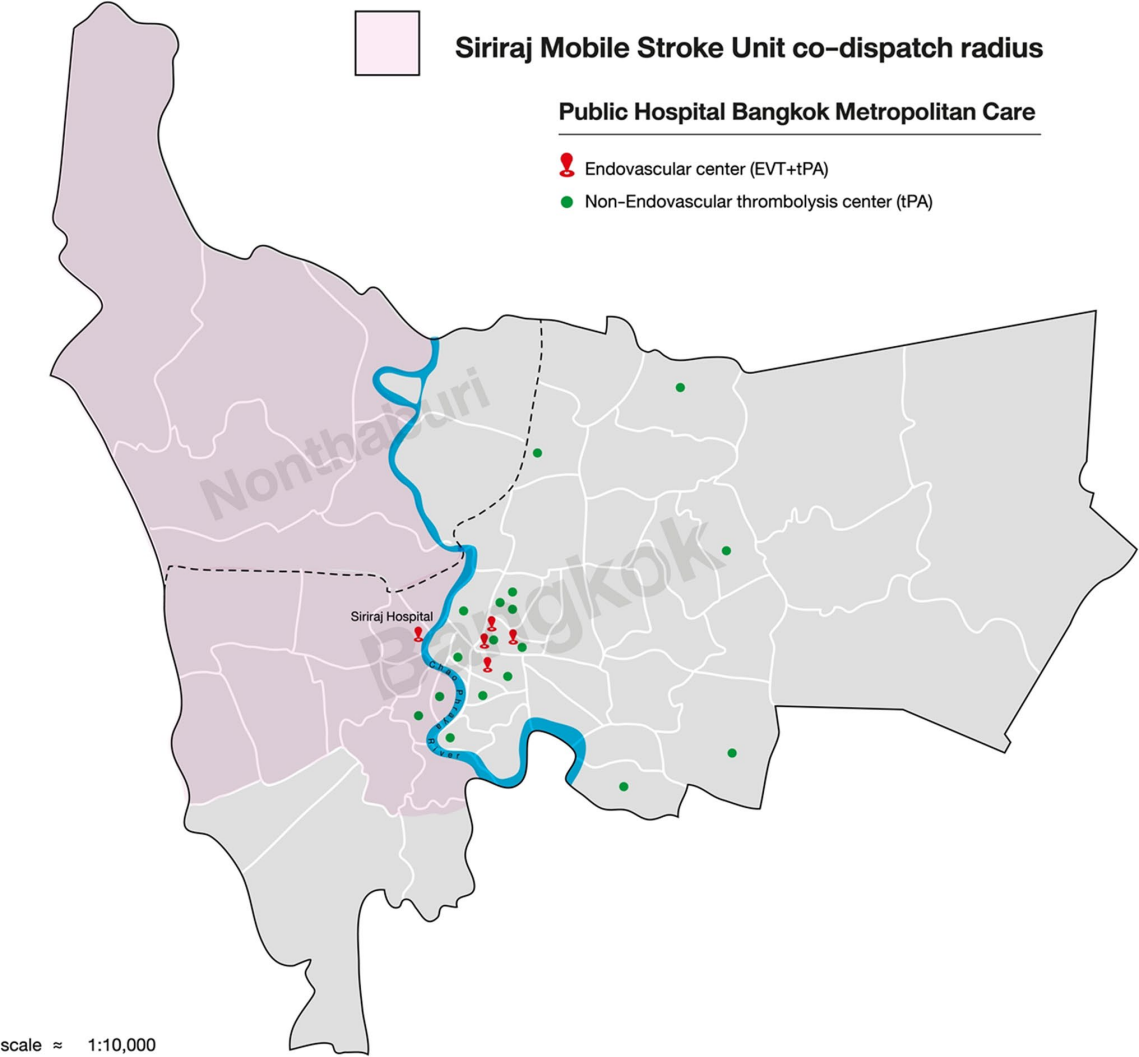
Mobile stroke unit catchment area

Bangkok is the capital city of Thailand with a population of 5.7 million in 2021 [11]. The city occupies 1568.7 km^2^ (605.7 square miles). It represents the paradigm of difficult timely access to therapies in large cities (e.g., due to traffic delays, blocked main streets to stroke centers). The city is divided by its main river, the Chaophraya River. On the east side, there are 20 teaching hospitals serving 3.9 million population (4 large university hospitals with 24/7 tPA and EVT facilities and another 16 public teaching hospitals with only intravenous (IV) tPA protocol availability). On the west side; however, there is only one university hospital, the Siriraj Hospital, Mahidol University, with 24/7 tPA and EVT protocol serving the population of 1.8 million in an area of 450 km^2^ (Fig. 1). The Siriraj Hospital is a tertiary care center with over 2000 beds. Hospital statistics showed that acute stroke admission has been increasing from 869 cases in 2010 to 1382 cases in 2020. Emergency Medical Acts and Protocols from health authorities for acute stroke calls are in place since 2008 [12]. The acronym FAST (Facial drooping, Arm weakness, Speech difficulties and Time) has been used by national health authorities to educate the public on detecting symptoms of a stroke. Specifically, patients with any stroke symptoms within 4.5 h are encouraged to call an emergency number 1669 (similar to 911 in North America). An EMS dispatcher triaged those calls according to the symptoms, onset, and location.

#### The Siriraj ED acute stroke fast track protocol

The stroke code is activated in patients presented at the ED with acute neurological deficits within 4.5 h after the onset. The protocol consists of a rapid neurological assessment by neurology residents and/or stroke fellows, acute stroke lab (complete blood count, POCT (point-of-care testing) for glucose, and INR (international normalized ratio) and brain imaging studies (computerized tomography, CT), CT angiogram (CTA), and/or CT perfusion (CTP) using the RAPID software. Intravenous t-PA for eligible patients is administered in the CT room, whereas endovascular treatment (EVT) is administered in the Siriraj angiographic suite.

#### The mobile stroke unit (MSU)

**Fig. 2:**
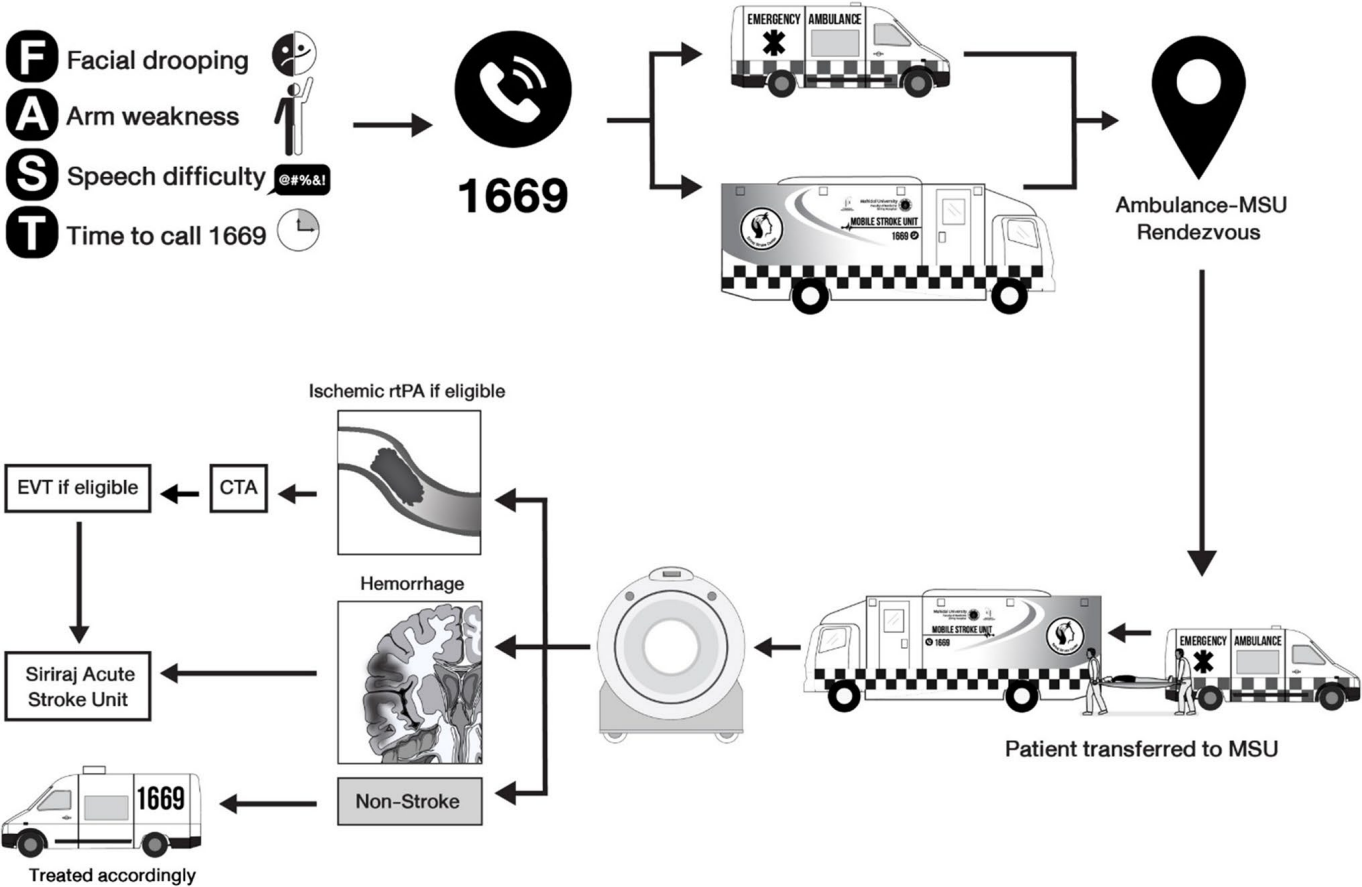
Mobile stroke unit operational flow

The first Siriraj MSU was established in June 2018 to provide 24/7 operation for acute stroke care initially for patients living in the west side of Bangkok Metropolitan and Nonthaburi province. (Fig. 1) The MSU is activated for suspected stroke within 4.5 h. The MSU protocol (Fig. 2) is operated by 2 vehicles: the regular ambulance and the MSU. Both units are notified by the EMS dispatcher at the same time. The regular ambulance picks up patient from home and brings patient to the predetermined rendezvous (area). The MSU, staffed with a physician (with training in stroke fast track protocol), a stroke nurse, a CT technician, and a driver is based at the Siriraj Hospital. Once the two vehicles meet at the midpoint area, patients are transferred to the MSU which is equipped with a portable CT machine and point of care testing for blood glucose and INR. After the completion of history taking, vital signs evaluation and rapid neurological assessment (NIHSS evaluation) by on board physician, a non-contrast CT scan of the brain is performed. The MSU has connected the stroke expert(s) from the Siriraj Stroke Center to the patient via a telemedicine connection. Once the consultation process is completed, the MSU physician starts treatment with IV tPA, if indicated. Other medication(s) can be given if necessary, i.e., IV antihypertensive medication to bring blood pressure down prior to IV thrombolysis administration. Treatment is started on the MSU, while the patient is being transferred to the Siriraj Stroke Center. Once patients arrive, they are sent for CT and multiphase CTA for EVT eligibility evaluation. If the patients are candidates for EVT, they are transferred to the angiographic suite. Following reperfusion therapies, patients are admitted to the Siriraj Acute Stroke Unit. Standardized procedures post-reperfusion were in place, including a follow-up non-contrast CT brain at 24 h to detect asymptomatic intracranial hemorrhage.

#### Data collection and definitions

Patients’ demographic data including age, sex, initial stroke severity assessed by NIHSS score, and comorbidities such as hypertension (HT), diabetes mellitus (DM), dyslipidemia (DLP), atrial fibrillation (AF), and history of prior stroke were collected by chart review. Process time measures including onset (time when patients experienced symptom(s) or time last seen normal, if unknown onset), door time (defined as ED arrival time or the time that the MSU and the regular ambulance meet at the rendezvous), CT, and needle time (defined as time when the first non-contrast CT brain is performed and iv tPA bolus dose is given, respectively) were collected from medical records. Puncture time was determined when the groin was first punctured during the EVT procedure. A favorable outcome was defined as a modified Rankin Scale (mRS) of 0 to 2 at 3 months.

Telephone follow up with structured questions was allowed for mRS evaluation.

The study protocol was approved by the ethical review committee of the Faculty of Medicine, Siriraj Hospital, Mahidol University, Bangkok, Thailand (SIRB protocol No 877/2562, IRB 3).

### Outcome measures

The primary time process measure was door to needle (DN) time. The primary clinical outcome measure was the rate of independency at 3 months (mRS 0–2). Secondary outcomes were the proportion of patients who received reperfusion treatment (tPA and/or EVT), door-to-CT (DCT), and door-to-puncture (DP) time. Access to acute reperfusion treatment including IV tPA and/or EVT and 3-month mRS were compared among the three groups.

### Statistical analysis

Baseline characteristics of patients were presented as frequency, percentage, mean (± SD), and median (IQR). Statistical comparisons of baseline characteristics were performed according to mode of hospital arrival using one-way ANOVA test for continuous variable and Pearson chi-square test for categorical variables. The mean difference statistics were used to measures the absolute difference between the mean process time among three groups (MSU, EMS, and walk-in). One-way ANOVA and Tukey’s post hoc test with a 95% confidence interval were used to evaluate a significant difference of process times between the three groups. The association of clinical outcome measures by 3-month mRS and mode of hospital arrival was analyzed using logistic regression to obtain the odds ratio (OR) with 95% CI. Disabilities at 3 months were assessed by the mRS 0–2 with adjustment by age, sex, baseline NIHSS, comorbidities (prior stroke, the presence of diabetes, hypertension, dyslipidemia, and atrial fibrillation (AF)), DN time, and reperfusion therapies (e.g., receiving tPA and/or EVT). We were also interested in evaluating if the potential benefit of MSU was related with shorter door-to-needle times. As a result, we included this variable in the model. All statistical analysis was 2-tailed test, and *p*-values of < 0.05 were considered significant. We used STATA 14 (College Station, TX: StataCorp LP) to conduct all analyses.

## Results

**Fig. 3:**
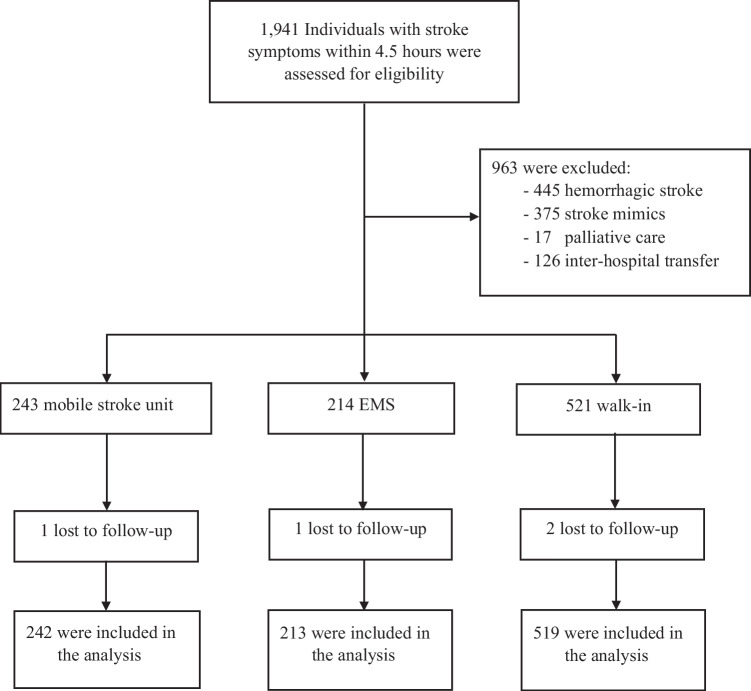
Participant flowchart

Of 1941 eligible participants, 963 were excluded for not meeting the inclusion criteria [445 (28.4%) with hemorrhagic stroke, 375 (38.9%) with stroke mimics, 126 (13.1%) transferred from another institution, 17 (1.8%) on palliative care]. A total of 978 patients (MSU = 243, EMS = 214, and walk-in = 521) met the inclusion criteria during the study period (Fig. 3). The mean age was 66 (± 14); 47.9% were women. There was no significant difference in patient’s demographics amongst groups except for the presence of AF, prior history of stroke, and baseline NIHSS. The EMS patients tended to be more severe (mean NIHSS = 9 (± 8), 12 (± 8),7 (± 7.1); *p* < 0.001) in MSU, EMS, and walk-in, respectively. AF was found to be the most prevalent in the EMS (MSU = 20.9%, EMS = 22.9%, walk-in = 15.3%; *p* = 0.028), while prior history of stroke was the most frequent among the walk-in group (MSU = 12.3%, EMS = 11.2%, walk-in = 18.8%; *p* = 0.01). Further details are shown in Table 1.

**Table 1:** Patients’ characteristics

	Total *n* = 978	MSU *n* = 243	EMS *n* = 214	Walk-in *n* = 521	*P*-value^a^
Age (year)					0.148^b^
Median (IQR)	66 (56, 76)	66 (54, 75)	67 ( 56, 77)	66 (57, 76)	
Mean (SD)	66 (14)	65 (13.9)	66 (14.3)	66 (13.6)	
Age > 65 years, *n* (%)	509 (52)	125 (51.4)	113 (52.8)	271 (52)	0.958
Male sex, n (%)	510 (52.1)	138 (56.8)	119 (55.6)	253 (48.6)	0.055
Initial NIHSS					< 0.001^b^
Median (IQR)	6 (3, 15)	6 (2, 14)	13 (5, 19)	4 (2, 11)	
Mean (SD)	9 (8)	9 (8)	12 (8)	7 (7.1)	
Initial NIHSS					< 0.001
NIHSS 0–9	617 (63.1)	152 (62.6)	89 (41.6)	376 (72.2)	
NIHSS ≥ 10	361 (36.9)	91 (37.4)	125 (58.4)	145 (27.8)	
Comorbidities, n (%)					
Hypertension	653(66.8)	174(71.6)	138(64.5)	341(65.4)	0.176
Diabetes	325(33.2)	80(32.9)	61(28.5)	184(35.3)	0.203
Dyslipidemia	412(42.1)	108(44.4)	85(39.7)	219(42)	0.593
AF	180(18.4)	51(20.9)	49(22.9)	80(15.3)	0.028
Prior stroke	152(15.5)	30(12.3)	24(11.2)	98(18.8)	0.01
All patients					
Onset-to-door (minutes)					0.185^b^
Median (IQR)	110 (70, 170)	118 (83, 165)	105 (65, 175)	110 (61, 173)	
Mean (SD)	122 (65)	128 (56)	120 (65)	119 (69)	
Min, max	[4, 270]	[38, 268]	[15, 267]	[4, 270]	
Door-to-CT (minutes)					< 0.001^b^
Median (IQR)	11 (8, 16)	6 (4, 8)	12 (9, 15)	13 (11, 18)	
Mean (SD)	15 (14)	7 (4)	16 (13)	18 (16)	
Min, max	[1, 110]	[1, 39]	[3, 91]	[3, 110]	
Patients receiving thrombolysis					
Onset-to-needle time (minutes)					0.31^b^
Total, *n* (%)	362 (100)	136 (37.6)	89 (24.6)	137 (37.8)	
Median (IQR)	124 (94, 172)	126 (85, 178)	127 (83, 168)	121 (85, 270)	
Mean (SD)	135 (54)	140 (52)	132 (57)	130 (60)	
Min, max	[35, 277]	[52, 264]	[39, 265]	[35, 277]	
Door-to-needle time (minutes)					< 0.001^b^
Total, *n* (%)	362 (100)	136 (37.6)	89 (24.6)	137 (37.8)	
Median (IQR)	25 (19, 32)	18 (15, 24)	26 (21, 31)	31 (25, 40)	
Mean (SD)	28 (14)	20 (7)	29 (13)	35 (16)	
Min, max	[7, 114]	[7, 40]	[17, 98]	[15, 114]	
Patients receiving thrombectomy					
Onset-to-puncture time (minutes)					0.08^b^
Total, *n* (%)	150 (100)	31 (20.8)	53 (35.6)	66 (44)	
Median (IQR)	181 (143, 240)	216 (180, 254)	165 (135, 205)	172 (136, 250)	
Mean (SD)	196 (71)	218 (47)	182 (65)	196 (82)	
Min, max	[83, 476]	[147, 365]	[92, 377]	[83, 476]	
Door-to-puncture time (minutes)					< 0.001^b^
Total, *n* (%)	150 (100)	31 (20.8)	53 (35.6)	66 (44)	
Median (IQR)	80 (70, 98)	68 (63, 75)	80 (68, 95)	92 (76, 113)	
Mean (SD)	90 (36)	73 (19)	86 (33)	101 (42)	
Min, max	[35, 302]	[35, 127]	[53, 262]	[54, 302]	
3-mo modified Rankin Score (mRS)					0.001
Total, n(%)	974 (100)	242 (24.8)	213 (21.9)	519 (53.3)	
mRS 0–2	602 (61.8)	170 (70.3)	108 (50.7)	324 (62.4)	
mRS 3–4	156 (16)	32 (13.2)	41 (19.3)	83 (16)	
mRS 5–6	216 (22.2)	40 (16.5)	64 (30)	112 (21.6)	

*MSU* mobile stroke unit, *EMS* emergency medical services, *walk-in* walk-in patients, *IQR* interquartile range, *SD* standard deviation, *NIHSS* National Institutes of Health Stroke Scale.

^a^*P* values for Pearson chi-square test of difference between MSU, EMS, and walk-in.

^b^One-way ANOVA test for difference mean between three groups MSU, EMS, and walk-in.

### Process time measures

There was a significant reduction of key process time measures including DCT, DN, and DP time in the MSU when compared to the usual care (walk-in and EMS groups) as shown in Table 1. Mean ± SD DCT was significantly reduced from 18 ± 16 to 16 ± 13 min in the walk-in and EMS, respectively, to 7 ± 4 min in the MSU group (*p* < 0.001). Patients in the MSU group had the shortest DN and DP time compared to the EMS and walk-in group (mean DN ± SD was 20 ± 7 min in the MSU versus 35 ± 16 and 29 ± 13 min in the walk-in and EMS, respectively, *p* < 0.001). Mean DP + SD was 73 ± 19 min in the MSU versus 101 ± 42 and 86 ± 33 min in the walk-in and EMS, respectively, *p* < 0.001. Further details are presented in Table 1.

### Access to reperfusion therapy

Overall, 412 (42.1%) participants received reperfusion therapy during the study period. Of those, 262 (26.8%) received only IV tPA, 50 (5.1%) underwent only EVT, and 100 (10.2%) received both IV tPA and EVT. After adjusting for age, sex, initial NIHSS, prior stroke, and AF, participants in the MSU were more likely to received reperfusion treatment (IV tPA and/or EVT) compared to the walk-in and EMS groups (odds ratio, 2.83; 95%CI, 2.01–3.99 for MSU vs walk-in and odds ratio, 2.18; 95% CI, 1.43–3.32 for MSU and EMS). More details are provided in Table 2.

**Table 2:** Unadjusted and adjusted odd ratio for reperfusion therapy and ICH outcomes by mode of hospital arrival

Outcome		n (%)	Unadjusted		Adjusted	
			OR^a^ (95% CI)	*P*-value	OR^b^ (95% CI)	*P*-value
Reperfusion therapy						
tPA and/or EVT (*n* = 412)	Walk-in	164/521 (31.5)	1		1	
	EMS	110/214 (51.4)	2.3 (1.66, 3.19)	< 0.001	1.3 (0.9, 1.89)	0.166
	MSU	138/243 (56.8)	2.86 (2.09, 3.92)	< 0.001	2.83 (2.01, 3.99)	< 0.001
	EMS		1		1	
	Walk-in		0.43 (0.31, 0.6)	< 0.001	0.77 (0.53, 1.12)	0.166
	MSU		1.42 (0.86, 1.79)	0.249	2.18 (1.43, 3.32)	< 0.001
ICH post received reperfusion therapy**						
All ICH (*n* = 73)	Walk-in	31/164 (18.9)	1		1	
	EMS	19/109 (17.4)	0.9 (0.48, 1.7)	0.758	0.67 (0.34, 1.31)	0.24
	MSU	23/137 (16.8)	0.86 (0.48, 1.57)	0.634	0.88 (0.47, 1.65)	0.697
	EMS		1		1	
	Walk-in		1.1 (0.59, 2.07)	0.758	1.5 (0.76, 2.94)	0.24
	MSU		0.96 (0.49, 1.86)	0.894	1.32 (0.65, 2.7)	0.442
SICH (*n* = 22)	Walk-in	9/164 (5.5)	1		1	
	EMS	4/109 (3.7)	1.03 (0.51, 2.09)	0.935	0.62 (0.18, 2.14)	0.448
	MSU	9/137 (6.6)	0.73 (0.36, 1.5)	0.396	1.19 (0.45, 3.18)	0.715
	EMS		1		1	
	Walk-in		1.52 (0.46, 5.08)	0.492	1.62 (0.47, 5.62)	0.448
	MSU		1.85 (0.52, 6.16)	0.319	1.94 (0.56, 6.79)	0.299

*OR* odd ratio, *CI* confidential interval, *tPA* tissue plasminogen activator, *EVT* endovascular treatment, *ICH* intracerebral hemorrhage, *SICH* symptomatic intracerebral hemorrhage. Data are numbers (percentage).

^a^Crude (unadjusted) odds ratio using logistic regression analysis.

^b^Adjusted for age, sex, initial NIHSS, prior stroke, and atrial fibrillation using multiple logistic regression analysis.

**Missing data of ICH post received intravenous tPA = 2 (EMS = 1, MSU = 1).

### Intracerebral hemorrhage post-reperfusion therapy

Overall, 73 (17.7%) patients experienced intracerebral hemorrhage (ICH) within 24 h after receiving reperfusion treatment (tPA and/or EVT). Symptomatic ICH occurred in 22 (5.3%) patients (MSU = 6.6%, EMS = 3.7%, walk-in = 5.5%). There was no significant difference in the incidence of symptomatic and asymptomatic ICH among 3 groups in both adjusted and unadjusted models (Table 2).

### Clinical outcomes at 3 months

**Fig. 4:**
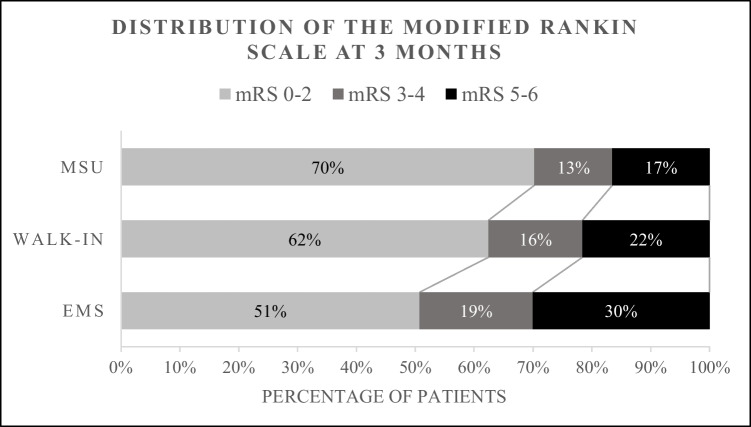
A 3-month modified Rankin Scale distribution

Participants in the MSU group had the highest rates of favorable outcome at 3-month (mRS = 0–2) compared to their counterparts (MSU = 70.3%, walk-in = 62.4%, EMS = 50.7%; *p* = 0.001) (Table 1 and Fig. 4). After adjusting for age, sex, baseline NIHSS, prior stroke, the presence of diabetes, hypertension, dyslipidemia, atrial fibrillation, reperfusion therapies and onset to needle time, participants in the MSU were two-fold more likely of achieving favorable outcome at 3 months when compared to the EMS (odds ratio, 2.15; 95% CI, 1.39–3.32) (Table 3; model B). Interestingly, age, NIHSS, and MSU were the overwhelming factors associated favorable outcome, whereas onset to needle time loss its significance. Further details are demonstrated in Table 3.

**Table 3:** Factors associated with good outcome (mRS 0–2)

	Unadjusted		Adjusted	
	OR^a^ (95% CI)	*P*-value	OR^b^ (95% CI)	*P*-value
Mode of hospital arrival				
EMS	Ref		Ref	
Walk-in	1.61 (1.17, 2.23)	0.003	1.3 (0.91, 1.88)	0.154
MSU	2.29 (1.56, 3.35)	< 0.001	2.15 (1.39, 3.32)	< 0.001
Reperfusion therapy				
None	Ref		Ref	
tPA	0.84 (0.62, 1.14)	0.260	0.96 (0.67, 1.37)	0.822
EVT	0.58 (0.33, 1.04)	0.067	1.2 (0.62, 2.32)	0.585
Combined tPA and EVT	1.03 (0.66, 1.61)	0.886	2.08 (1.25, 3.53)	0.005
Age > 65 years	0.26 (0.2, 0.34)	< 0.001	0.28 (0.2, 0.38)	< 0.001
Male, ref: female	1.47 (1.13, 1.89)	0.004	1.12 (0.84, 1.49)	0.444
Initial NIHSS				
NIHSS ≥ 10	Ref		Ref	
NIHSS 0–9	3.17 (2.42, 4.16)	< 0.001	3.45 (2.47, 4.82)	< 0.001
Atrial fibrillation	0.68 (0.49, 0.94)	0.02	1.15 (0.79, 1.69)	0.451
Hypertension	0.71 (0.54, 0.94)	0.018	0.97 (0.7,1.32)	0.829
Previous stroke	0.69 (0.49, 0.97)	0.037	0.74 (0.49, 1.09)	0.125
Diabetes	0.82 (0.63, 1.08)	0.161	N/A	N/A
Dyslipidemia	0.99 (0.76, 1.28)	0.936	N/A	N/A
Onset to needle time	1.001 (0.99, 1.004)	0.584	N/A	N/A

^a^Crude (unadjusted) odds ratio using logistic regression analysis.

^b^Multiple logistic regression analysis used to evaluate factors associated with good outcomes.

## Discussion

Mobile stroke units are becoming the current paradigm of stroke care delivery to shorten the time to reperfusion therapies and improve patient’s independency. To the best of our knowledge, this is the first MSU study prospectively conducted in a developing country comparing time process measures and stroke outcome among three different AIS groups according to mode of hospital arrival: MSU, EMS, and walk-in. Our study showed that AIS patients living in underserved setting receiving care by MSU had shorter DCT, DN, and DP times; higher probability of receiving reperfusion treatment(s); and double the chance of achieving independency at 3 months when compared to conventional ambulance and walk-in.

Comparing with previous MSU studies from developed countries, several findings were replicated concerning time performance measures in acute stroke management [8, 9, 13].We found a significant reduction of DCT, DN, and DP time in the MSU groups. The MSU patients was associated with a 31% and 42.9% reduction in an average DN and a reduction of 15.1% and 27.7% in an average DP time when compared to those in the EMS and walk-in, respectively.

Of importance, there was no significant difference in onset-to-door (OD) time among the three groups. This reflects a major improvement of the implementation of the MSU as patients from more remote areas usually arriving by EMS or as walk-in would have had a longer delay (average from historical controls of 160 min) in arriving to a stroke center or to a closer non-stroke center with no reperfusion therapies. Furthermore, the finding that OD was similar between MSU and the EMS and “walk-in” groups also emphasizes the need to increase stroke awareness and improve the response time of EMS in regions and countries where the implementation of MSU for underserved areas is not realistic.

The fact that MSUs provided a faster and more direct access to neuroimaging studies and vascular neurologist consultation via telemedicine resulting in a streamline workflow can explain a higher rate of reperfusion treatment. This finding was confirmed in our study with a nearly threefold (OR 2.83; 95% CI 2.01–3.99) and two-fold (OR 2.18; 95% CI 1.43–3.32) likelihood of receiving reperfusion treatment when compared to walk-in and EMS, respectively. However, a higher incidence rate of symptomatic ICH post-IV tPA (5.3%) was found when compare to prior trials [8, 9]. This finding is aligned with previous reports from studies in Asiatic populations [14, 15]. Genetic factors, tPA dose, and pharmacogenetics may explain the higher risk of symptomatic ICH post-thrombolysis among Asians when compared to the Caucasian population [16].

Although the AIS treatment guidelines [17] recommended that using EMS in acute stroke was independently associated with earlier ED arrival, faster ED evaluation, and higher proportion of reperfusion therapy, AIS patients in the EMS group only accounted for 21.9% in our study. Similar findings were demonstrated in several studies from low-middle income countries in which 10–25% of acute stroke patients were transported to hospitals by EMS [18–21] From our experiences in Thailand, where a majority of acute stroke patients are self-referred or walk-in, the EMS is usually called when the patients are more severe. This practice probably is true in other developing countries, and it could explain the unbalance of stroke severity between the 3 groups in our study in which the initial NIHSS was significantly higher in the EMS. However, after adjusting for any potential confounders, the MSU was shown to be significantly associated with the highest chance of getting a good neurological recovery (mRS 0–2) at 3 months (adjusted OR 2.15 (1.39, 3.32). To further explain our study results, we believed that the benefits of having MSU service were not entirely from the reduction in time metrics. There were several potential advantages that the MSU paradigm of care is providing over usual care for patients with an AIS: (i) shortening the time required for reperfusion treatment, (ii) getting immediate access to telemedicine consultation with vascular neurologist, (iii) faster diagnosis of a large vessel occlusion, (iv) prompt initiation of reperfusion therapy with tPA, and increasing the likelihood of receiving EVT alone or as add-on therapy. All of these advantages contributed to the new working paradigm in providing the most time efficient acute stroke care in the field and finally resulting in higher probability of achieving independency.

Our study has several limitations: (i) as our study was not a randomized-controlled trial, it is subject to the limitations of observational studies; for example, more severe stroke patients used EMS as a mode of transportation, more patients experienced prior stroke were enrolled in the walk-in, and more atrial fibrillation found in the EMS. (ii) Although the same enrollment criteria were used, we were not able to exclude the possibility of biases related to the mode of arrival. (iii) We cannot rule out the possibility of residual confounders that might remain despite a comprehensive adjustment. (iv) The exact distance between home and stroke center were not collected. Therefore, given the nature of our study, we cannot provide a clear explanation or infer causation.

Despite the limitations, our study should be seen as a proof-of-concept regarding the successful implementation of a new paradigm of stroke care (MSUs) in a country with limited resources. We had near complete (99.6%) follow-up data at 3 months. Finally, we applied a pragmatic approach as the study design was based on real-world practice by enrolling participants who followed the activation of the code stroke protocol either when they arrived at the ED or at the MSU. Limited information is available on real-world data from the developing world.

## Conclusion

Pre-hospital diagnosis and treatment of AIS patients using an MSU significantly shortened DCT, DN, and DP time; increased the likelihood of receiving reperfusion therapies; and improved stroke outcomes by reducing disability at 3 months when compared to standard care (EMS and walk-in). The MSU model is an innovative and evolving concept to improve acute stroke management, especially in underserved highly populated areas with delay access to tertiary stroke centers. Prospective studies are needed to determine whether or not the MSU paradigm is more cost-effective than usual care.
